# The Core-Targeted RRM2 Gene of Berberine Hydrochloride Promotes Breast Cancer Cell Migration and Invasion via the Epithelial–Mesenchymal Transition

**DOI:** 10.3390/ph16010042

**Published:** 2022-12-28

**Authors:** Jiaming He, Qiang Wei, Rong Jiang, Tiankuo Luan, Shuang He, Ruijin Lu, Hang Xu, Jianhua Ran, Jing Li, Dilong Chen

**Affiliations:** 1 Laboratory of Stem Cells and Tissue Engineering, Department of Histology and Embryology, College of Basic Medicine, Chongqing Medical University, Chongqing 400016, China; 2 Department of Laboratory Medicine, The First Affiliated Hospital of Chongqing Medical University, Chongqing 400016, China; 3 Neuroscience Research Center, College of Basic Medicine, Chongqing Medical University, Chongqing 400016, China; 4 Chongqing Key Laboratory of Development and Utilization of Genuine Medicinal Materials in Three Gorges Reservoir Area, Chongqing Three Gorges Medical College, Chongqing 404120, China

**Keywords:** berberine hydrochloride, breast cancer, RRM2, epithelial–mesenchymal transition, bioinformatics

## Abstract

Berberine hydrochloride (BBR) could inhibit the proliferation, migration, and invasion of various cancer cells. As the only enzyme for the de novo synthesis of ribonucleotides, RRM2 is closely related to the development of tumorigenesis. However, not much is currently known about the functional roles of RRM2 in breast cancer (BRCA), and whether BBR regulates the migration and invasion of BRCA cells by regulating the expression of RRM2 remains to be determined. We study the effects of BBR on BRCA cell proliferation in vitro and tumorigenesis in vivo by using colony formation assays, EdU assays, and xenograft models. Transcriptome sequencing, the random forest algorithm, and KEGG analysis were utilized to explore the therapeutic target genes and relative pathways. The expression of RRM2 in BRCA patients was analyzed with The Cancer Genome Atlas (TCGA) dataset, the GEPIA website tool, the Gene Expression Omnibus (GEO) database, and the UALCAN database. The survival probability of BRCA patients could be predicted by survival curve and nomogram analysis. Molecular docking was used to explore the affinity between BBR and potential targets. Gain- and loss-of-function methods were employed to explore the biological process in RRM2 participants. We comprehensively investigated the pharmacological characteristics of BBR on BRCA cell lines and discovered that BBR could inhibit the proliferation of BRCA cells in vitro and in vivo. Combining transcriptome sequencing and KEGG analysis, we found that BBR mainly affected the biological behavior of BRCA cells via HIF-1α and AMPK signal pathways. Additionally, by using bioinformatics and molecular docking, we demonstrated that RRM2 plays an oncogenic role in BRCA samples and that it acts as the hub gene of BBR on BRCA cells. Knockdown and overexpression studies indicated that RRM2 promoted BRCA cell migration as well as invasion in vitro by affecting the epithelial-to-mesenchymal transition (EMT). Our study demonstrated the significance of BBR regulating HIF-1α and AMPK signaling pathways in BRCA cells. Moreover, we revealed the carcinogenic role and potential mechanism of RRM2 as a core regulatory factor of BBR in BRCA in controlling BRCA invasion, migration, and EMT, suggesting that RRM2 may be a therapeutic target and prognostic biomarker for BRCA therapy.

## 1. Introduction

BRCA is the most common malignant tumor in women and the fifth leading cause of cancer mortality worldwide, with approximately 685,000 deaths yearly [1,2]. BRCA is highly heterogeneous; based on molecular and histological evidence, BRCA could be categorized into three groups. BRCA expresses hormone receptors (estrogen receptor (ER+) or progesterone receptor (PR+)). BRCA also expresses human epidermal receptor 2 (HER2+) and triple-negative breast cancer (TNBC) (ER−, PR−, and HER2−) [3]. The treatment strategies and risk profiles of different types of patients usually require personalization. In addition to primary surgical resection, patients can combine it with hormonal therapy, radiotherapy, or chemotherapy [4]. In addition, the accumulated evidence confirms that natural products have good efficacy in treating BRCA [5].

BBR, a well-known isoquinoline alkaloid, and its chemically pure form, were first isolated from *H. canadensis* (goldenseal) in 1917 [6]. To date, a comprehensive pharmacological study of BBR has been conducted, making it one of the best-studied natural products. Moreover, BBR showed good antitumor activity. Several types of cancer cells are directly affected by it [7,8,9,10]. However, as with many natural therapeutic products, there is a lack of high-quality scientific evidence regarding the core pathways and targets of BBR. The application of transcriptome sequencing comprehensively explored the characteristics of changes in transcription levels in drug-induced experimental models [11,12]. However, the transcriptomics data of BBR acting on BRCA cells are lacking.

Ribonucleotide reductase (RNR) is the only enzyme that catalyzes the de novo formation of deoxyribonucleotides [13]. It is a critical enzyme in DNA synthesis and plays an indispensable role in DNA replication and repair in cell cycle progression [14]. RNR consists of two subunits: the large subunit ribonucleotide reductase M1 (RRM1) and the small subunit (RRM2 or RRM2B). Interestingly, RRM2 is generally highly expressed in cancers [15]. It has been demonstrated that the overexpression of RRM2 is associated with drug and chemotherapy resistance [16,17]. Moreover, RRM2 silencing inhibited the progression of various tumor cells [18,19,20]. Moreover, several lncRNAs are involved in the advancement of BRCA cells by affecting the downstream expression of RRM2 [21,22]. Accumulating evidence revealed that RRM2 could be considered a tumor promoter and target for cancer therapy [23,24]. However, very little is known about the effects of RRM2 on malignant biological behaviors in BRCA.

This study aims to investigate the functional role and underlying mechanism of BBR in treating BRCA cells. The core pathways HIF-1α and AMPK of BBR were obtained by transcriptome sequencing. Moreover, we identified the core target RRM2 of BBR using multiple databases and random forest algorithms, and its increased expression was related to the worse survival rate of BRCA patients. BRCA cells with knockouts of RRM2 exhibit a marked reduction in migration and invasion capabilities. Additionally, RRM2 plays a critical role in metastasis and EMT. Our study provides novel insights into the role of BBR in regulating BRCA cell progression and identifies RRM2 as a potential therapeutic target.

## 2. Results

### 2.1. BBR Inhibits the Proliferation of BRCA Cells In Vitro and In Vivo

**Figure 1:**
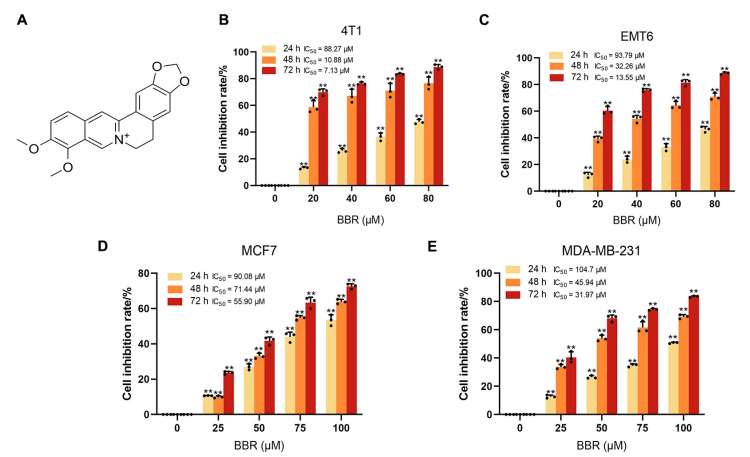
Inhibitory effects of BBR on BRCA cells. The chemical structure of BBR (**A**); 4T1 (**B**), EMT6 (**C**), MCF7 (**D**), and MDA-MB-231 (**E**) cells were treated with various concentrations of BBR for 24, 48, and 72 h, respectively. ** *p* < 0.01 vs. control. Data represent the means ± SD of three independent experiments.

**Figure 2:**
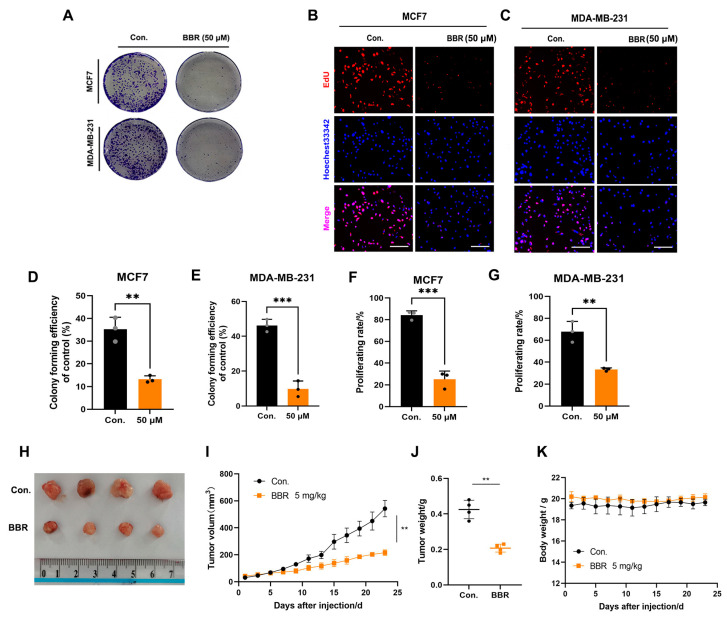
BBR inhibits the proliferation of BRCA cells in vitro and in vivo. The colony formation of MCF7 and MDA-MB-231 cells exposed, with or without BBR (**A**). MCF7 (**B**) and MDA-MB-231 (**C**) cells were incubated with BBR for 24 h, and cell proliferation rates were observed by EdU assay. Statistical analysis of colony formation and EdU experiment. Scale bars: 10 μm (**D**–**G**). ** *p* < 0.01, *** *p* < 0.001. Data represent the means ± SD of three independent experiments. BBR inhibited xenograft tumor size and tumor weight (**H**–**J**). No data differences were observed in the mouse body weight (**K**).

BBR is an isoquinoline alkaloid with good antitumor activity (Figure 1A). To explore the biological function of BBR in BRCA, two mouse BRCA cells (4T1 and EMT6) and two human BRCA cells (MCF7 and MDA-MB-231) were treated with BBR at gradient concentrations. Four BRCA cells were significantly inhibited by BBR in a dose- and time-dependent manner (Figure 1B–E). The half-maximal inhibitory concentrations (IC_50_) of 24 h BBR treatment were close to 88.27 μm, 93.79 μm, 90.08 μm, and 104.7 μm for 4T1, EMT6, MCF7, and MDA-MB-231, respectively. Then, we used the human species’ BRCA cells for further studies. Moreover, it was observed that BBR significantly suppressed the colony formation and proliferating rates of MCF7 and MDA-MB-231 cells, respectively (Figure 2A–G). To examine the effect of BBR on BRCA growth in vivo, MDA-MB-231 cells were subcutaneously inoculated into the BALB/c nude mice. The intraperitoneal injection of BBR significantly inhibited tumor growth and weight in tumor models (Figure 2H–J). Moreover, BBR did not affect the body weight of mice (Figure 2K). Meanwhile, the HE-staining analysis of liver and kidney sections showed no significant changes between the two groups (Figure S1). Collectively, BBR could inhibit the growth of BRCA cells in vitro and in vivo.

### 2.2. Signaling Pathway Enrichment of BBR Based on RNA-Seq

**Figure 3:**
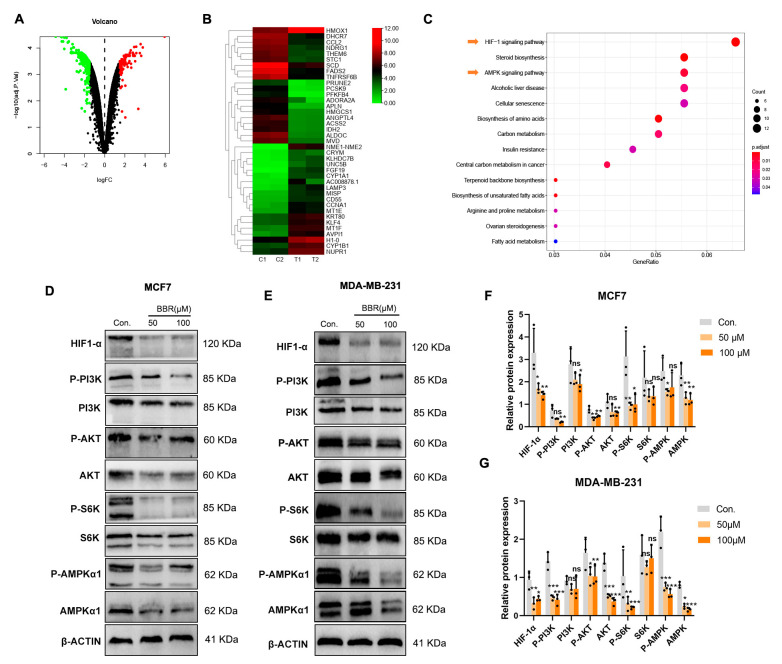
Signaling pathway enrichment of BBR based on RNA-seq. The volcano map shows DEGs (**A**). The heat map shows the top 20 upregulated and downregulated genes (C1, and C2: control group; T1 and T2: BBR treatment group; red, upregulated genes; green, downregulated genes) (**B**). KEGG demonstrates pathways for DEG enrichment in transcriptome sequencing (**C**). Western blot was used to demonstrate the changes in related pathways’ genes at the translation level in MCF7 and MDA-MB-231 (**D**–**G**) cells. ns: not statistically significant, * *p* < 0.05, ** *p* < 0.01, *** *p* < 0.001. Data represent the means ± SD of three independent experiments.

To explore the molecular mechanism of BBR in treating BRCA cells, the gene expression profiling of the BBR treatment group and control group was obtained by RNA-seq. In general, by using |LogFC| ≥ 1.5 and *p*-value < 0.05, 406 DEGs were identified, including 107 upregulated genes and 299 downregulated genes (Figure 3A). To confirm the data acquired by microarray, a random subset of upregulated genes (*n* = 10) and downregulated genes (*n* = 10) was chosen for RT-qPCR analyses, and the results showed that PCR fold changes correlated with the microarray data (Figure S2, Table S2). The top 20 upregulated and downregulated genes are shown in the heat map (Figure 3B). Then, the KEGG results confirmed the DEG enrichment with the HIF-1α and AMPK signaling pathways after BBR treatment (Figure 3C). The Western blot assay was applied in MCF7 and MDA-MB-231 cells to analyze the protein expression levels of the related signaling pathways, and AMPKα1, HIF-1α, PI3K, and AKT were down. Moreover, the phosphorylation of AMPKα1(Ser485), PI3K(Tyr458), AKT(Ser473), and S6K(Thr389) proteins also decreased (Figure 3D–G). These pieces of evidence suggested that BBR can target multiple oncogenic signaling pathways.

### 2.3. Random Forest Screening for DEGs

**Figure 4:**
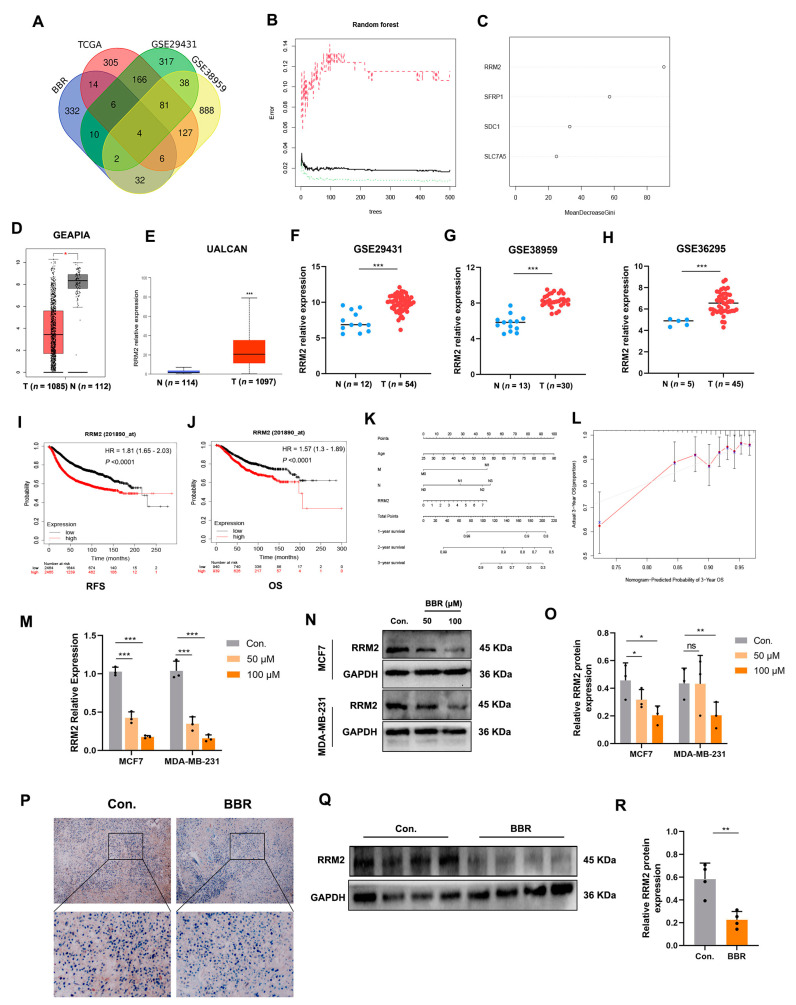
RRM2 as a potential regulator in BBR-treated BRCA cells. Venn diagrams depict DEGs associated with clinical significance and BBR targeting information in different transcriptome matrices (**A**). The influence of the number of decision trees on the error rate. The x-axis represents the number of decision trees, and the y-axis indicates the error rate (**B**). Results of the Gini coefficient method in the RF algorithm (**C**). Results of the Gini coefficient method in the RF classifier. The gene (RRM2) ranked in the top list according to the prognostic importance was chosen for further analyses. Multiple transcripts showed that RRM2 was highly expressed in BRCA tissues (**D**–**H**). The survival curves of RFS and OS (**I**,**J**). The nomogram for predicting the 3-year OS of BRCA patients (**K**). The calibration curve for the OS nomogram model (**L**). The ideal nomogram approximates the tilt of a gray line. rt-PCR and Western blot indicated that RRM2 expression was downregulated by BBR in MCF7 and MDA-MB-231 (**M**–**O**). Immunoblotting and Western blotting confirmed that RRM2 expression decreased in xenograft models (**P**–**R**). * *p* < 0.05, ** *p* < 0.01, *** *p* < 0.001. Data represent the means ± SD of three independent experiments. Representative images show high or low expressions of RRM2 in MDA-MB-231 xenograft tumor tissues. Scale bars: 2 μm (**O**).

TCGA-BRCA, GSE29431, and GSE58959 datasets were derived from clinical breast cancer patients and normal samples. The DEGs obtained from these transcriptome data were considered to be meaningful genes for clinical patients. The intersection of these three datasets with the BBR treatment transcriptome matrix was used to further obtain the DEGs. Moreover, the results shown in the Venn diagram included RRM2, SFRP1, SDC1, and SLC7A5 (Figure 4A). These genes were considered as potential targets for BBR intervention in breast cancer patients. Next, we input the four DEGs into the random forest (RF), a machine learning algorithm. We performed a recurrent random forest classification for all possible numbers among the four variables and calculated the average error rate of the model. The number of variables was as small as possible, and the out-of-band error was as low as possible. Referring to the relationship plot between the model error and the number of decision trees (Figure 4B), we selected 300 trees as the parameter of the final model, which showed a stable error in the model. In the process of constructing the random forest model, the variable importance of the output results (Gini coefficient method) was measured from the perspective of decreasing accuracy and decreasing mean squared error. Figure 4C shows that, among the four variables, RRM2 was the most important, followed by SFRP1, SDC1, and SLC7A5. RRM2 expression was also decreased by BBR in RNA-seq (Figure S3). In the TCGA and GEO database for BRCA, the Spearman’s correlation analysis revealed the strongest positive correlation between HIF-1α and RRM2 (Figure S4). Therefore, RRM2 was selected for in-depth investigations.

### 2.4. RRM2 as a Potential Regulator in BBR-Treated BRCA Cells

We used the GEPIA and UALCAN databases to confirm that RRM2 is overexpressed in BRCA patients compared to in normal samples (Figure 4D,E). In addition, we extracted three BRCA transcriptome matrices from the GEO database (GSE29431, GSE38959, and GSE36295). RRM2 was also highly expressed in BRCA samples (Figure 4F–H). Moreover, the survival curve in the K-Mplot database also indicated that patients with high expressions of RRM2 had worse recurrence-free survival (RFS) and overall survival (OS) (Figure 4I,J). Univariate and multivariate Cox regression analyses of TCGA-BRCA data revealed that RRM2 expression was significantly correlated with age, tumor stage, N, and M stage (Table 1). Then, we constructed a nomogram based on the multivariate Cox regression results of age, N, M, and RRM2 expression to predict the 3-year survival probability (Figure 4K). According to the contribution degree of each influencing factor in the model relative to the outcome variable (the size of the regression coefficient), each value level of each influencing factor was scored. The total points were obtained by adding the scores of each part. Finally, the predictive value of the individual outcome event was calculated with the function conversion relationship between the total points and the probability of the outcome event. Clinicians could evaluate the prognostic risk of newly admitted patients with long-term statistical patient points. The calibration curve shows that the nomogram can produce sufficient efficiency (Figure 4L). Moreover, the rt-PCR and Western blot showed that BBR could inhibit RRM2 expressions in MCF7 and MDA-MB-231 cells (Figure 4M–O). IHC and Western blot results indicated that RRM2 expression decreased in MDA-MB-231 tumor xenograft models treated with BBR (Figure 4P–R). It suggests that RRM2 could be a potential therapeutic target for BBR and an independent prognostic factor in BRCA patients.

### 2.5. Molecular Docking

**Figure 5:**
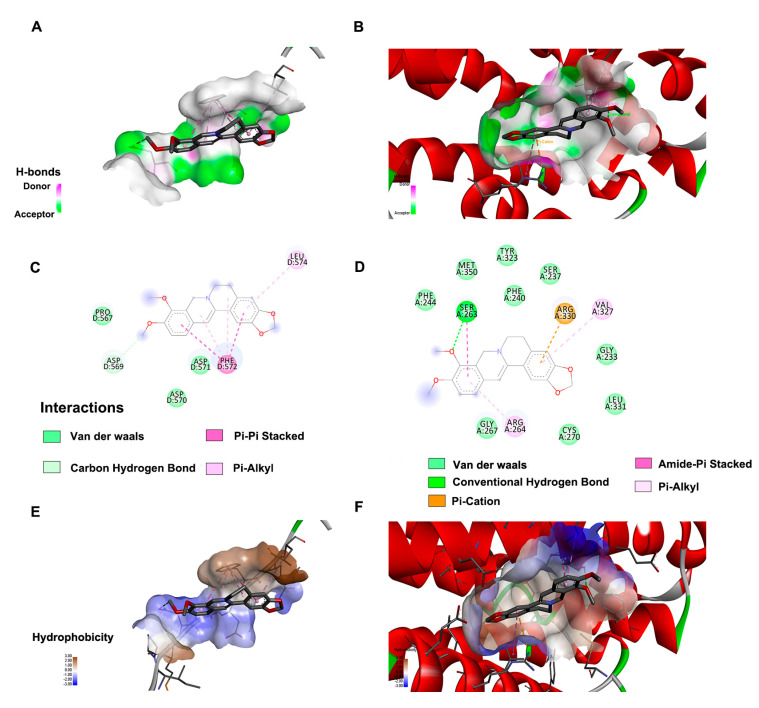
Molecular docking. Three-dimensional visualization of BBR docking results with HIF-1α and RRM2 (**A**,**B**); 2D BBR docking results with HIF-1α and RRM2 (**C**,**D**). The hydrophobic surface of HIF-1α and RRM2 interacts with BBR; the brown and blue colors represent hydrophobicity and hydrophily, respectively (**E**,**F**).

Based on the top rank of KEGG and the results of screening targeted genes, HIF−1α and RRM2 were considered the significant genes for BBR therapy. We propose that BBR regulates these targets by inhibiting their gene expression or directly blocking binding sites at the translational level. Therefore, we used Autodock Vina software for docking at the molecular level (Figure 5A−D). The energy affinity of BBR-RRM2 was −7.7 kcal/mol, and the forces involved were van der Waals forces, carbon−hydrogen bonds, pi−pi stacking, and pi−alkyl hydrophobic forces. According to the 2D schematic of the interaction (Figure 5C), BBR interacts with Pro567, Asp569, Asp570, Asp571, Phe572, and Leu574. Furthermore, four hydrophilic interactions (one van der Waals force and three carbon−hydrogen bonds) and five hydrophobic interactions were found (two pi−pi stacked and three pi−alkyl) between BBR and Leu574 and Phe572. Then, this was followed by BBR-HIF-1α (−5.9 kcal/mol). Moreover, the docking aminos were Gly233, Ser237, Phe240, Phe244, Ser263, Arg264, Gly267, Cys270, Tyr323, Val323, Val327, Arg330, Leu331, and Met350 (Figure 5D). There were ten hydrophilic interactions (nine van der Waals forces and one carbon−hydrogen bond) and four hydrophobic interactions (one pi−cation, two pi−alkyl hydrophobic forces, and one amide−pi stacked) between BBR and Arg330 (3.83 Å), Arg264 (4.52 Å), Val327 (5.36 Å), and Ser263 (3.91 Å). It is worth noting that the hydrogen bond distance was relatively long, which suggests that it is not very strong (Figure 2CD). Notably, it was also shown in Figure 5C−F that the hydrophobic effect of BBR is closely related to the presence of substituted benzene and isoquinoline rings. All of these amino acids may be possible sites of interactions between the protein and BBR. These sites remind us of whether it is useful to add a benzene ring or an isoquinoline ring to enhance the effects of BBR. These results suggest that both hydrogen bonding and hydrophobic interactions play an important role in the binding of the BBR to the HIF−1α and RRM2. Moreover, BBR may be a promising inhibitor for the RRM2 and HIF-1α protein.

### 2.6. RRM2 Promotes Migration and Invasion of BRCA Cells In Vitro

**Figure 6:**
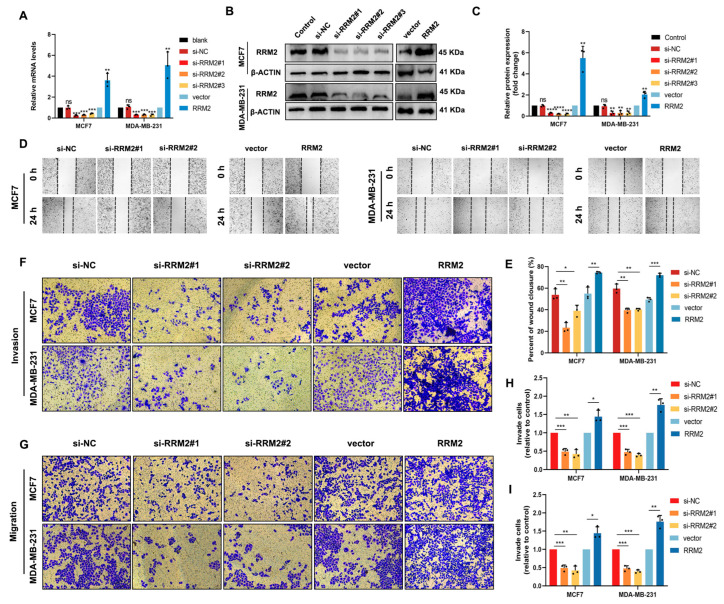
RRM2 promotes migration and invasion of BRCA cells in vitro. Knockdown and overexpression of RRM2 in MCF7 and MDA-MB-231 cells were confirmed by RT-qPCR (**A**) and Western blot (**B**,**C**). Wound healing (**D**,**E**) and transwell assays (**F**–**I**) show the mobility and invasiveness of BRCA cells after the knockdown or overexpression of RRM2. ns: not statistically significant, * *p* < 0.05, ** *p* < 0.01, *** *p* < 0.001, **** *p* < 0.0001. Data represent the means ± SD of three independent experiments.

Our results of multivariate Cox regression in Table 1 indicate that RRM2 expression is strongly correlated with metastasis. An essential initial step in cancer metastasis is the migration and invasion of cancer cells into surrounding tissues and vessels [25]. To determine whether RRM2 induces the invasion and migration ability of BRCA cells, we transfected small interference sequences targeting RRM2 into MCF7 and MDA-MB-231 cell lines or established cell lines overexpressing RRM2. The RT-qPCR results showed that RRM2 was successfully silenced or overexpressed at the mRNA level (Figure 6A). The efficiency of the knockdown and overexpression was also validated by Western blot at the protein level (Figure 6B,C). In addition, the wound healing analysis of MCF7 and MDA-MB-231 cells showed that the knockdown of RRM2 inhibited migration, whereas the overexpression of RRM2 had the opposite effect (Figure 6D,E). Transwell analyses further confirmed that silencing RRM2 attenuated the migration and invasion, whereas RRM2 overexpression enhanced these abilities in MCF7 and MDA-MB-231 cells (Figure 6F–I). Taken together, the above studies indicated that RRM2 promotes the migration and invasion abilities of BRCA cells.

### 2.7. RRM2 Regulates the EMT Program of BRCA Cells In Vitro

**Figure 7:**
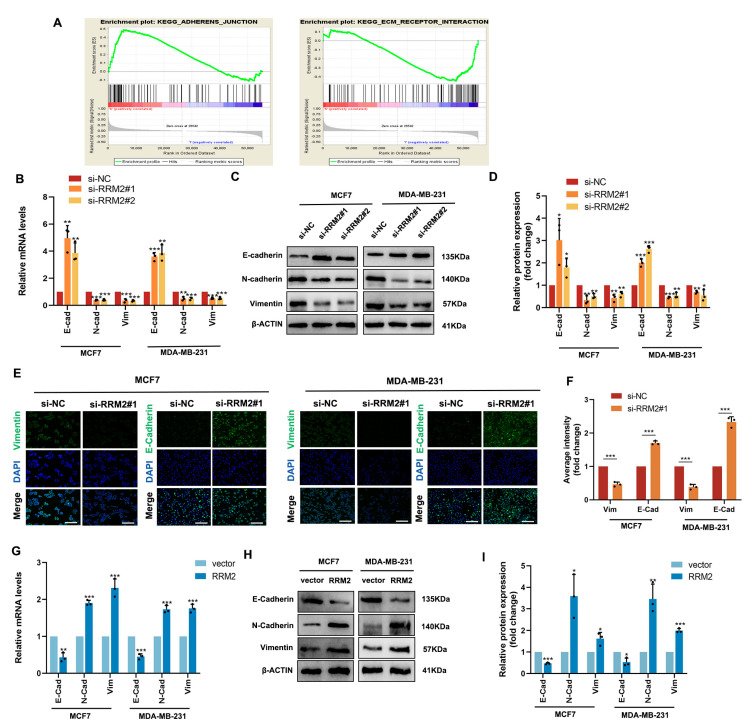
RRM2 regulates the EMT program of BRCA cells in vitro. The GSEA results show the RRM2 enrichment pathway (**A**). The levels of E-cadherin, N-cadherin, and vimentin expression were identified with RT-qPCR (**B**) and Western blotting (**C**,**D**) after being transfected with si-NC or si-RNAs in MCF7 and MDA-MB-231. The expression and distribution of E-cadherin and vimentin were identified by immunofluorescence (**E**,**F**). Scale bars: 50 μm. MCF7 and MDA-MB-231 were transduced with a corresponding control vector and RRM2 plasmids. The expression levels of E-cadherin, N-cadherin, and vimentin were identified with RT-qPCR (**G**) and Western blot (**H**,**I**). * *p* < 0.05, ** *p* < 0.01, *** *p* < 0.001. Data represent the means ± SD of three independent experiments.

**Figure 8:**
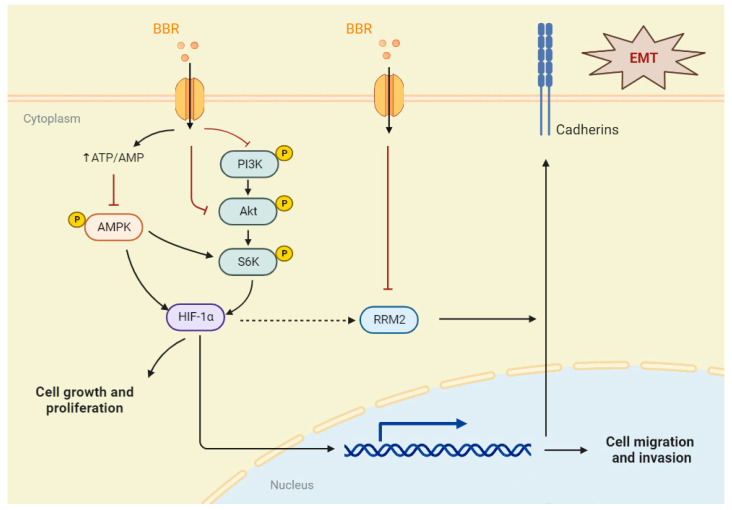
Schematic diagram of the core pathway and target of BBR on BRCA cells. A graphic illustration of the proposed mechanism in this study.

A GSEA algorithm was applied to the TCGA-BRCA data to further investigate the potential biological functions of RRM2 in BRCA. As shown in Figure 7A, the diagram is divided into three parts. The first part involves the following: The green line at the top is a line chart of the gene enrichment score. The vertical axis is the corresponding enrichment score, and there is a peak in the line chart, which also represents the maximum enrichment fraction of this gene set. The second part involves the barcode−like part in the middle, and each vertical bar pair should be a gene under the gene set: red represents the RRM2 high expression group, and blue represents the RRM2 low expression group. The third part involves (the bottom) the distribution of ranking values for all genes, and the ordinate is the value of the number of gene sorts. The results showed that KEGG ADHERENS JUNCTIONS was significantly enriched in the RRM2 high group, and the KEGG ECM RECEPTOR INTERACTION was skewed toward the low expression of RRM2 (Figure 7A). This evidence suggests that RRM2 may be involved in the EMT process. During tumor metastasis, EMT is a critical step [26]. Subsequently, we used functional assays to determine the role of RRM2 in BRCA cells undergoing EMT. RT−qPCR and Western blot analyses in MCF7 and MDA-MB-231 cells revealed that the silence of RRM2 decreased E-cadherin expression, but it increased N-cadherin and vimentin expression (Figure 7B–D). In addition, immunofluorescence staining further confirmed that silencing RRM2 resulted in a loss of vimentin expression, while E-cadherin’s average intensity was upregulated, suggesting that BRCA can modulate the EMT program (Figure 7E,F). Then, we examined MCF7 and MDA-MB-231 cells that were transduced with vector and RRM2 plasmids and found that E-cadherin was indeed downregulated, while N-cadherin and vimentin were upregulated both at the gene and protein levels (Figure 7G–I). Above all, it suggests that RRM2 plays a vital role in modulating the EMT program in BRCA cells (Figure 8).

## 3. Discussion

Most BRCA patients do not have metastases at diagnoses. However, the median overall survival of patients with metastatic BRCA is approximately 5 years or less [3]. Although surgery is an effective treatment for localized tumors, there are limited options for treating metastatic BRCA. Therefore, it is essential to understand the underlying molecular mechanisms involved in BRCA metastasis and to identify effective targets for therapeutic strategies.

As a natural molecule, BBR has been demonstrated in the bit proliferation and metastasis of various cancers [7,8,9,10,27]. However, previous research on BBR in BRCA cells was limited to verifying known oncogenic pathways and targets, and the critical information for drug development was missing. In this study, a comprehensive strategy based on transcriptomics and bioinformatics was established to investigate the regulatory characteristics of BBR on BRCA cells. Based on DEGs, KEGG results indicated that the HIF-1α and AMPK signaling pathways were the key pathways regulated by BBR. The HIF-1α and AMPK signaling pathways are evolutionarily conserved survival mechanisms responding to two fundamental stresses, oxygen deprivation and/or energy deficiency. This also proves the importance of the efficient development of BBR.

Active hypoxia-inducible factors (HIFs) are composed of the constitutively expressed HIF-1β subunit, an O2-dependent HIFα isoform, and essential cofactors [28]. Moreover, in BRCA, HIF-1α is the predominantly expressed isoform of HIFα [28]. Studies have confirmed that HIF-1α is a driver of aggressive treatment resistance and poor prognosis in BRCA [28]. Genetic alterations common in BRCA and the hyperactivation of PI3K/Akt/mTOR or MAPK pathways increase HIF-1α transcriptional translation or stability independent of O_2_ levels [28,29]. Furthermore, in vitro and in vivo studies have shown that activating transcriptional programs controlled by HIF-1α could promote both mobile and aggressive BRCA phenotypes [30]. Kuo et al. found that BBR can be used as an adjuvant therapeutic agent by targeting the Akt pathway and inhibiting the metastatic ability of BRCA cells by downregulating the expressions of MMP2 and MMP9 [7]. According to this study, we examined the effects of BBR on the pathway at the translational level and found that the phosphorylation of the PI3K (Tyr458), AKT (Ser473), and S6K (Thr389) was inhibited. In addition, the expressions of PI3K and AKT were also decreased. It revealed that BBR could inhibit the expression of HIF-1α by targeting the PI3K/Akt/S6K pathway, thereby reducing the proliferation, migration, and invasion of BRCA cells.

The AMP-activated protein kinase (AMPK) is a serine/threonine protein kinase [31,32,33,34,35,36,37,38,39]. The phosphorylation of AMPK-mediated TSC2 and Raptor proteins regulates mTOR signal expression with negative feedback relative to the suppressed downstream S6K [39]. The activation or inhibition of AMPK is closely related to the development of canceled proliferation and metastasis [3]. Lee et al. revealed that BBR with no more than 6 h of treatment could transiently activate AMPKα1 and inhibits AKT [33]. However, we found that BBR could decrease the phosphorylation of AMPKα1 (Ser485) and AMPKα1 after 24 h of treatment. Interestingly, compared with the low-concentration BBR treatment group, the P-AMPKα1/AMPKα1 rate of the IC_50_ group is upregulated in MCF7. Therefore, we suggest that BBR can target the reduction in AMPKα1 and that there is a time- and dose-dependent association between BBR and P-AMPKα1. Previous research studies reported that the pharmacological or genetic inhibition of AMPK diminished HIF-1α activations [34,35]. We have also demonstrated the inhibitory effect of BBR on HIF-1α. It indicated that BBR could also directly affect the proliferation and invasion of BRCA cells by inhibiting the AMPKα1/HIF-1α pathway or reducing the negative feedback mechanism of AMPKα1 regulating S6K phosphorylation.

Currently, abnormal gene expression has been viewed as one of the factors contributing to the occurrence and development of BRCA, and some abnormal gene expressions may be useful as biomarkers in the diagnosis and prognosis of BRCA. As a result, we analyzed the BBR, TCGA-BRCA, GSE38959, and GSE29431 datasets to find DEGs associated with BRCA pathogenesis. The coincident DEGs in the four transcriptomes were considered influential and clinically significant for BBR therapy. Then, we combine the advantages of machine-learning techniques to improve the predictive power of finding drug targets. RF offers exceptional performance in processing multifeature data with great accuracy and precision [36]. The results of RF classifier screening suggest that RRM2 is the most well-studied gene. In addition, the molecular docking results showed a strong affinity of BBR to HIF-1α and RRM2 via hydrophobicity and hydrophily bonds. This strong affinity is closely related to the benzene and isoquinoline rings in the berberine hydrochloride structure. Moreover, the above results confirmed the accuracy of our KEGG results and RF algorithm prediction.

Recently, studies have shown that the LncRNA/miRNA axis regulates RRM2 expression. For instance, the AFAP1-AS1/miR-139-5p axis controls tumor growth and attenuates chemotherapy resistance by targeting RRM2 [37]. Moreover, the SNHG16/miR-30a/RRM2 axis also accelerates BRCA cell proliferation and invasion, while miR-4500 could downregulate RRM2 and inhibit BRCA cell proliferation, invasion, and migration via suppressing the MAPK signaling pathway [38,39]. Moreover, RRM2 has been included in several bioinformatic-based clinical prognostic models [40,41]. In addition, a single gene bioinformatics study and one clinical pathology research study indicated that RRM2 expression could help in evaluating the outcome of BRCA patients [42,43]. In our study, we extracted the transcriptomic matrix and pathological features of BRCA samples from the TCGA database and found that RRM2 expression was associated with tumor metastasis by multivariate Cox regression. Functional analysis revealed that overexpressing RRM2 has the opposite effect to siRRM2 in terms of enhancing the capacity for migration and invasion. However, the mechanism by which RRM2 upregulated the migration and invasion of the BRCA cells remains to be determined.

Via the GSEA algorithm, we found that patients with high expressions of RRM2 were positively correlated with the adherens junction, and they were negatively correlated with the extracellular matrix (ECM) receptor’s interaction. These results bring us to the possible involvement of RRM2 in the EMT process. EMT has been proven as a critical factor in tumorigenesis and metastasis [25]. The activation of EMT leads to the disruption of intercellular junctions, the degradation of underlying basement membranes, and the reorganization of ECM. It is molecularly characterized by a loss of cell adhesion and epithelial markers, such as E-cadherin. Moreover, it leads to the acquisition of enhanced invasions and mesenchymal markers, including N-cadherin and vimentin [26]. Wang et al. indicated that the overexpression of RRM2 was correlated with the EMT marker Slug in oral squamous cell carcinoma tissues [44]. Yang et al. revealed that RRM2 overexpression promoted retinoblastoma cell migration, invasion, and EMT [45]. In pancreatic cancer, a natural small molecule, astaxanthin, acts via the HIF-1α/STAT3 axis to mediate RRM2, regulating the gemcitabine-induced EMT phenotype [46]. In BRCA cells, we first demonstrated that the knockdown of RRM2 increased E-cadherin expression and decreased N-cadherin and vimentin expression, while the overexpression of RRM2 had the opposite effect. Meanwhile, we demonstrated a strong positive correlation between HIF-1α and RRM2 expression using multiple clinical datasets. However, the specific regulatory mechanism between HIF-1α and RRM2 remains unknown. In cervical cancer, the upregulation of RRM2 could promote cervical carcinogenesis via ROS-ERK1/2-HIF-1α-VEGF-induced angiogenesis [47]. Yan et al. found that HIF-1α can regulate the expression of RRM2 by activating the STAT3 transcription factor in pancreatic cancer cells [46]. However, the details of the regulation between HIF-1α and RRM2 still need further study in breast cancer. The above findings and our data indicated that BBR inhibits HIF-1α via the AMPK and PI3K/AKT/S6K pathways, and it also reduces the expression of RRM2, causing the inhibition of epithelial–mesenchymal transitions in BRCA cells.

There are also some limitations in this study. First, the clinical BRCA samples are deficient because access to tissue is invariably difficult. Second, in vivo experiments with the stable transfection of RRM2 will be carried out later. Collectively, further investigation is required to gain a better understanding of the detailed mechanisms.

## 4. Materials and Methods

### 4.1. Online Data

RNA-sequencing matrix and related clinical information regarding BRCA were derived from The Cancer Genome Atlas (TCGA) database, which contained 1109 BRCA cases and 113 normal cases (http://portal.gdc.cancer.gov (accessed on 2 March 2022)). The Gene Expression Omnibus (GEO) database was used to extract the RNA-seq data from clinical samples (http://www.ncbi.nlm.gov/geo/, accessed on 24 April 2022). UALCAN (http://ualcan.path.uab.edu/analysis, accessed on 28 April 2022) and GEPEA (http://gepia.cancer-pku.cn, accessed on 28 April 2022) databases were utilized to investigate the RRM2 expression between normal and tumor samples. Differentially expressed genes (DEGs) in online data were conducted using the R package of “limma” according to the criteria of the false discovery rate (FDR) < 0.05 and |LogFC| ≥ 1.5. The KMplot (http://kmplot.com/analysis/, accessed on 28 April 2022) database was utilized to obtain the survival curve.

### 4.2. Random Forest Algorithm

The random forest algorithm (RF) was performed using the R package “randomForest”. The gene included was considered a variable. By reducing accuracy and mean square errors, the variable importance of the output results was measured in this model [36]. The highest-ranked variables are what we consider core genes.

### 4.3. Nomogram Analysis

To examine the relationship between the expression of RRM2 and BRCA patients’ clinical characteristics, univariate and multivariate Cox regressions were conducted. Each variable was calculated using R’s “forestplot” package to determine its *p*-value, hazard ratio (HR), and 95% confidence interval (CI). Based on the results of the multivariate Cox regression, a nomogram was created to predict the 3-year overall survival. For each patient, one can calculate the risk of recurrence by summing up the points associated with each risk factor in the “rms” package of R. Afterwards, a calibration plot was created to test its predictive power.

### 4.4. GSEA Analysis

Signaling pathways significantly associated with RRM2 expression in TCGA-BRCA data were analyzed with GSEA 4.2.3 software (http://www.gsea-msigdb.org/gsea/index.jsp, accessed on 5 May 2022). The molecular signature dataset of c2.cp.kegg.1.Hs.symbols.gmt in GSEA 4.2.3 was used to identify enriched pathways between normal and BRCA samples. The criteria for significantly enriched pathways are as follows: *p*-value < 0.05, *q*-value < 0.25, and normalized enrichment score (NES) > 1 [48].

### 4.5. Molecular Docking

The structure of berberine was downloaded from Pubchem and drawn by using ChemDraw software, version: 14.0. The crystal structures of HIF-1α (ID: 1LQB) and RRM2 (ID: 3OLJ) were obtained from the RCSB PDB (http://www.rcsb.org/, accessed on 7 July 2022). The redundant components of the crystal structure were removed by PyMOL software. The interactions of berberine on HIF-1α and RRM2 were calculated by AutoDock Vina [49]. The 2D and 3D structures of the docking results were displayed by Discovery Studio. When the affinity energy is negative, the molecular proteins docked and cross-talked with each other spontaneously. Meanwhile, with lower energies, the molecular conformation was more stable.

### 4.6. Reagent and Antibodies

BBR (B21449, purity ≥ 98%) was purchased from Shanghai Yuanye Biotechnology Co., Ltd. (Shanghai, China), which was dissolved with dimethyl sulfoxide (DMSO) and stored at −20 °C. Primary antibodies against the proteins HIF-1α, AMPKα1, P-AMPKα1, PI3K, P-PI3K, AKT, P-AKT, S6K, P-S6K, and E-cadherin were purchased from Cell Signaling Technology (Danvers, MA, USA), while those against N-cadherin, vimentin, and RRM2 were purchased from Abclonal (Wuhan, China). The secondary antibodies, which were affinity-purified, anti-mouse, and anti-rabbit IgG, were obtained from CST. Table S1 contains detailed product numbers.

### 4.7. Cell Culture

Breast cancer cell lines 4T1, EMT6, MCF-7, and MDA-MB-231 were provided by Chongqing Key Laboratory of Stem Cells and Tissue Engineering. Cells were cultured with 8% fetal bovine serum (8%-FBS) RPMI 1640 medium (Gibco BRL, Dreieich, Germany) with 1% penicillin and streptomycin (C100C5, NCM Biotech, Suzhou, China) and grown at 5% CO_2_ and 37 °C.

### 4.8. Cell Viability Assay

Cell counting Kit-8 (CCK8, NCM Biotech, Suzhou, China) was used to estimate cell viability. Cells were seeded in 96-well plates at a density of 3 × 10^3^ cells per well. After cellular adherence, the cells were treated with BBR for 24, 48, and 72 h, respectively. Then, 10 μL CCK8 was added according to the instructions and incubated for 0.5 h. The absorbance was detected at 450 nm (Bio-RAD, Hercules, CA, USA).

### 4.9. Transcriptome Sequencing

Total RNA from MDA-MB-231 cells (control vs. BBR) was extracted using TRIzol (Takara, Maebashi, Japan). Then, the samples were stored at −80 °C and transcriptome sequencing technology was provided by Tsingke Biotechnology Co., Ltd. (Beijing, China). A significant amount of DEGs were selected based on this condition: *p* < 0.05 and |LogFC| ≥ 1.5. Analyses with respect to the Kyoto Encyclopedia of Genes and Genomes (KEGG) were performed using the R 4.0 package “ClusterProfiler” based on DEGs. The Venn diagram was drawn based on the DEGs in different matrices (http://www.bioinformatics.psb.ugent.be/webtools/Venn/, accessed on 28 April 2022).

### 4.10. Cell Colony Formation and 5-Ethynyl-2′-Deoxyuridine (EdU) Assay

MCF-7 and MDA-MB-231 cells in 6-well plates with 1 × 10^3^ cells per well were treated with or without BBR for 24 h. After 14 days, all plates were fixed with 4% paraformaldehyde (NCM Biotech, Suzhou, China) and stained with crystal violet staining solution (Beyotime, Shanghai, China). The EdU cell proliferation kit (Cellorlab, CX004, Shanghai, China) was also used to investigate the changes in cell proliferation according to the manufacturer’s instructions. Photographs were taken and calculated under a fluorescence microscope (Leica, Munich, Germany).

### 4.11. Wound-Healing Assay

MCF7 and MDA-MB-231 cells were inoculated into the 6-well plate. After the cells were fully grown, a straight line was scratched with a sterilized 200 μL head. Cells were incubated in 2% serum 1640. After 24 h, the field of view from each group was randomly selected for observation and photographed under an inverted microscope.

### 4.12. Transwell Assay

Migration and invasion were examined in a polycarbonate chamber (8 μm pore, Labselect, Hefei, China). MCF7 and MDA-MB-231 cells were resuspended in a 200 μL blank 1640 medium (2 × 10^5^ cells) and added to the upper chamber. Then, 20% FBS 1640 medium was added to the lower chamber. After 24 h, the cells were fixed and stained. The upper chamber of the polycarbonate chamber for the invasion assay was coated with Matrigel (082704, ABW, Shanghai, China), and the rest of the protocol was performed similarly to the migration assay. The number of cells in three randomly microscopic fields was selected for analysis (Leica, Munich, Germany).

### 4.13. Western Blot (WB) Assay

Western blot assay was carried out in BBR and functional experiments in MCF7 and MDA-MB-231 cells. The detailed procedures have been described in our previous study [50].

### 4.14. Immunofluorescence (IF) Staining

Cells were fixed with 4% paraformaldehyde at room temperature, permeabilization with 0.1% Triton X-100 (P0096, Beyotime, Shanghai, China), and, lastly, blocked with goat serum (AR0009, Boster, Wuhan, China). Then, the corresponding primary antibodies were added and incubated overnight at 4 °C. The following day, cells were incubated with vimentin (1:200) or E-cadherin (1:100) at room temperature for 1 h. Then, the nuclei were stained with DAPI (P0131, Beyotime, Shanghai, China). Photographs were captured with a fluorescence microscope (Leica, Munich, Germany).

### 4.15. Quantitative Real-Time PCR Assay (RT-qPCR)

mRNA and cDNA were carried out as described previously [50]. The 2^−ΔΔCt^ approach was utilized to assess relative expression levels, with GAPDH as an internal control. The primer sequences were listed in Table S2.

### 4.16. siRNA and Plasmids Transfection

A negative control siRNA and three independent siRNAs targeting RRM2 were purchased from GenePharma (Shanghai, China). The detailed sequences are shown in Table S2. Vector (P25093) and RRM2-expressing plasmids were purchased from Miaolingbio (Wuhan, China). Lipofectamine 3000 (Invitrogen, Carlsbad, CA, USA) was utilized for the transfection of cells with siRNAs (50 nM) and incubated for 6 h. After 48 h, cells were collected for subsequent experiments. Vector and RRM2-expressing plasmid (3 μg) were also transfected with Lipofectamine 3000. After 6 h, cells were changed to a fresh cell-culture medium for subsequent studies.

### 4.17. Xenograft Models in Nude Mice

The animal experiment procedures were authorized by the Animal Experiment Center of Chongqing Medical University (ID:20220944). BALB/c nude mice (female, 4–5 weeks old) were purchased from SJA Laboratory Animal Technology Co., Ltd. (Hunan, China). A total of 1 × 10^6^ MDA-MB-231 cells were subcutaneously injected into the right axilla of each mouse. Upon reaching a tumor size of approximately 40 mm^3^, mice were divided into two groups (four mice in every group). The experimental groups included the control group (saline, every 2 days) and the BBR group (5 mg/kg, once every 2 days, intraperitoneally). The mice were euthanized after 28 days, and tumors were excised, weighed, and embedded in paraffin. The tumor volume was calculated with the formula: Volume = 0.5 × (length × width^2^).

### 4.18. Immunohistochemistry (IHC) Analysis

Xenograft tumor tissues in paraffin sections were dewaxed and rehydrated. Next, endogenous peroxidase and nonspecific binding sites were blocked with 10% bovine serum albumin (AR0009, Boster, Wuhan, China) for 1 h. Then, rabbit anti-RRM2 (1:50, Abclonal, Wuhan, China) antibody was applied to all sections at 4 °C for 14 h. The following day, binding was conducted with the corresponding peroxidase-conjugated secondary antibody (A21020, Abbkine, Wuhan, China) and incubated at room temperature for 30 min. Then, the diaminobenzidine solution was used to detect the targeted antigen.

### 4.19. Statistical Analysis

All statistical analyses were conducted with GraphPad Prism 9 (San Diego, CA, USA). We applied a two-tailed Student’s *t* test to compare results between two different groups, and one-way ANOVA was conducted for multiple comparisons. Statistical significance was determined as values of *p* < 0.05. * *p* < 0.05, ** *p* < 0.01, *** *p* < 0.001, and **** *p* < 0.0001; ns: not statistically significant.

## 5. Conclusions

In summary, based on bioinformatics mining and experiments, our study reveals the critical role of HIF-1α and AMPK signaling pathways in the BBR treatment of BRCA cells. Moreover, we reported for the first time that RRM2 could promote BRCA cell migration and invasion via EMT. As a result of our findings, RRM2 may be helpful as a predictive biomarker in BRCA patients and as a potential target for antimetastatic interventions.

## Figures and Tables

**Table 1:** Univariate Cox-regression analysis and multivariate Cox-regression analysis of RRM2 expression and clinicopathologic variables in TCGA BRCA patients.s.

Variables	Univariate Analysis		Multivariate Analysis	
	HR (95%CI)	*p*-Value	HR (95%CI)	*p*-Value
Age	1.97 (1.39–2.81)	0.00 *	2.13 (1.47–3.08)	0.00 *
Gender	0.85 (0.12–6.13)	0.88	0.58 (0.08–4.21)	0.59
Tumor stage	2.70 (1.90–3.85)	0.00 *	1.88 (1.09–3.24)	0.02 *
T N M RRM2	1.87 (1.26–2.78) 2.21 (1.52–3.21) 6.54 (3.67–11.65) 1.10 (0.94–1.28)	0.00 * 0.00 * 0.00 * 0.25	0.94 (0.57–1.57) 1.64 (1.04–2.58) 2.86 (1.49–5.48) 1.17 (1.00–1.37)	0.81 0.03 * 0.00 * 0.05 *

Abbreviations: RRM2, ribonucleotide reductase regulatory subunit M2; HR, hazard ratio; CI, confidence interval. Variables: RRM2, high vs. low; age, ≥60 (y) vs. <60 (y); gender, male vs. female; tumor stage, stage I/II vs. III/IV; T, T1/T2 vs. T3/T4; M, M0 vs. M1; N, N0 vs. N1/N2/N3. * *p* < 0.05.

## Data Availability

The original contributions presented in this study are included in the article/Supplementary Materials. Further inquiries can be directed to the corresponding authors. TCGA and GEO belong to public databases. The patients involved in the database have obtained ethical approval. Users can download relevant data for free for research and publish relevant articles. TCGA and GEO databases utilized were approved by Institutional Review Board of The College of Basic Medical, Chongqing Medical University.

## Supplementary Materials

The following supporting information can be downloaded at: https://www.mdpi.com/article/10.3390/ph16010042/s1, Supplementary Figure S1: Representative HE images of liver and kidney in two groups, Figure S2: Validation of RNA-seq data, Figure S3: Relative expression of RRM2 in RNA-seq, Figure S4: Spearman correlation of RRM2 with HIF-1α in multiple RNA-seq. Table S1: Antibodies used in the experiments, Table S2: Primers used in the experiments.
