# Supplementary material for: The Core-Targeted RRM2 Gene of Berberine Hydrochloride Promotes Breast Cancer Cell Migration and Invasion via the Epithelial–Mesenchymal Transition
Source: Pharmaceuticals (Basel). 2022 Dec 28;16(1):42. doi: 10.3390/ph16010042 (PMC9861674; doi:10.3390/ph16010042)
Supplement: Supplementary file 1 [file pharmaceuticals-16-00042-s001.zip › Supplement_Figure.pdf]

SUPPLEMENTARY FIGURES

Figure S1

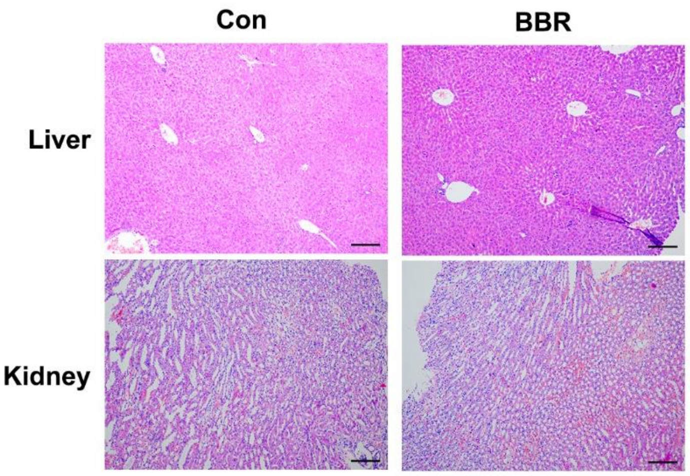

Figure S1. Representative HE images of liver and kidney in two groups.

Representative HE images of liver and kidney between control and berberine treating groups. Scale bars: 10 μm.

Figure S2

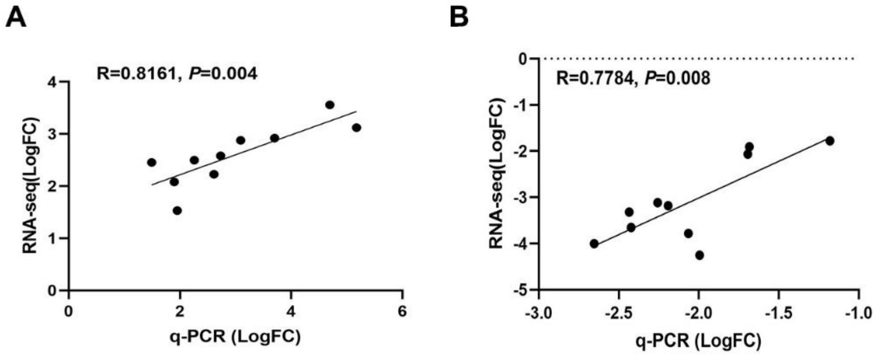

Figure S2. Validation of RNA-seq data.

RNA-seq data is validation by independent set of samples using q-PCR, Pearson correlation (R). Upgruated genes group(S2A) and downgraduated genes group(S2B).

Figure S3

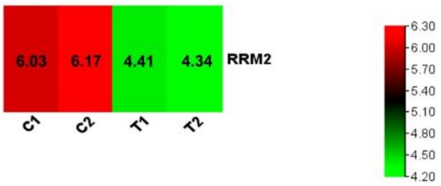

Figure S3. Relative expression of RRM2 in RNA-seq.

Relative expression of RRM2 in transcriptome sequencing between control group and BBR treatment group was shown in heat map. (C1, and C2: control group; T1 and T2: BBR treatment group; relative expression: Log（FPKM+1））

Figure S4

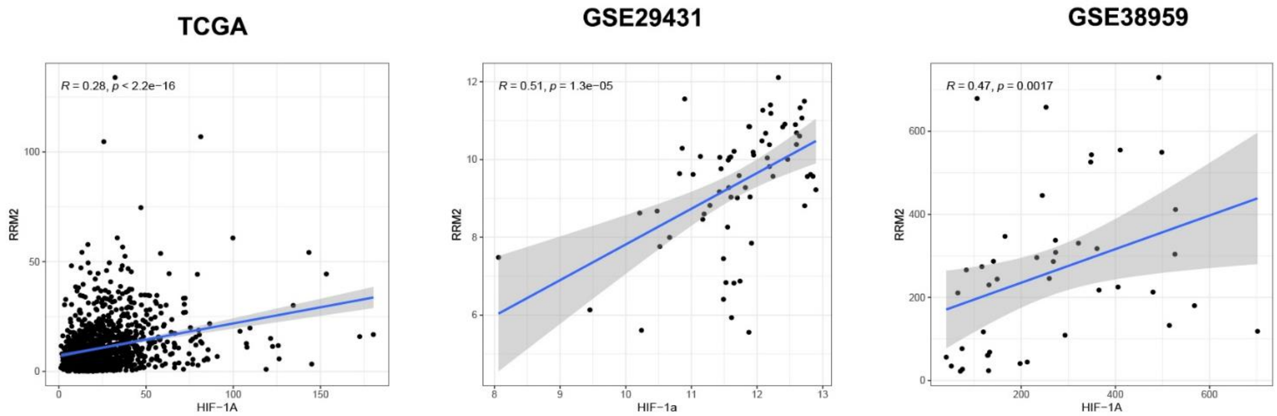

Figure S4. Spearman correlation of RRM2 with HIF-1α in multiple RNA-seq.

Spearman correlation of RRM2 with HIF-1α in the multiple RNA-seq for BRCA and normal samples. *P* value and Spearman's correlations are indicated.
