# Supplementary material for: The Core-Targeted RRM2 Gene of Berberine Hydrochloride Promotes Breast Cancer Cell Migration and Invasion via the Epithelial–Mesenchymal Transition
Source: Pharmaceuticals (Basel). 2022 Dec 28;16(1):42. doi: 10.3390/ph16010042 (PMC9861674; doi:10.3390/ph16010042)
Supplement: Supplementary file 1 [file pharmaceuticals-16-00042-s001.zip › Table_S1.pdf]

Table S1. Antibodies used in the experiments.

Antibody	Source	No. of Catalogue	Dilution		
			WB	IHC	IF
HIF-1α	Cell Signaling Technology, USA	79233	1:1000		
AMPKα1	Cell Signaling Technology, USA	2795	1:1000		
P-AMPKα1	Cell Signaling Technology, USA	2537	1:1000		
PI3K	Cell Signaling Technology, USA	4257	1:1000		
P-PI3K	Cell Signaling Technology, USA	17366	1:1000		
AKT	Cell Signaling Technology, USA	4691	1:1000		
P-AKT	Cell Signaling Technology, USA	4060	1:1000		
S6K	Cell Signaling Technology, USA	9202	1:1000		
P-S6K	Cell Signaling Technology, USA	9234	1:1000		
E-cadherin	Cell Signaling Technology, USA	14472	1:1000		1：100
N-cadherin	Abclonal, China	A0433	1:1000		
Vimentin	Abclonal, China	A11952	1:1000		1：200
RRM2	Abclonal, China	6241	1:1000	1：50	

Abbreviations: WB: Western blot; IHC: Immunohistochemistry; IF: Immunofluorescence
