# Supplementary material for: The Core-Targeted RRM2 Gene of Berberine Hydrochloride Promotes Breast Cancer Cell Migration and Invasion via the Epithelial–Mesenchymal Transition
Source: Pharmaceuticals (Basel). 2022 Dec 28;16(1):42. doi: 10.3390/ph16010042 (PMC9861674; doi:10.3390/ph16010042)
Supplement: Supplementary file 1 [file pharmaceuticals-16-00042-s001.zip › Table_S2.pdf]

**Table S2. Primers used in the experiments.**

| Gene name | | Primer sequence | Application |
|---|---|---|---|
| RRM2 | F | 5′-AGTGGAAGGCATTTTCTTTTCC-3′ | qRT-PCR |
| | R | 5′-GCAAAATCACAGTGTAAACCCT-3′ | |
| E-Cadherin | F | 5′-ATTTTTCCCTCGACACCCGAT-3′ | qRT-PCR |
| | R | 5′-TCCCAGGCGTAGACCAAGA-3′ | |
| N-Cadherin | F | 5’-TGCGGTACAGTGTAACTGGG-3′ | qRT-PCR |
| | R | 5’-GAAACCGGGCTATCTGCTCG-3′ | |
| Vimentin | F | 5’-AGTCCACTGAGTACCGGAGAC-3′ | qRT-PCR |
| | R | 5’-CATTTCACGCATCTGGCGTTC-3′ | |
| GAPDH | F | 5’-CAGGAGGCATTGCTGATGAT-3′ | qRT-PCR |
| | R | 5’-GAAGGCTGGGGCTCATTT-3′ | |
| si-NC | sense | 5’-UUCUCCGAACGUGUCACGUTT-3′ | si-RNA |
| | anti-sense | 5’-ACGUGACACGUUCGGAGAATT-3′ | |
| si-RRM2#1 | sense | 5'-CCAUCGAGUACCAUGAUAUTT-3' | si-RNA |
| | anti-sense | 5'-AUAUCAUGGUACUCGAUGGTT-3' | |
| si-RRM2#2 | sense | 5'-GGAGCGAUUUAGCCAAGAATT-3' | si-RNA |
| | anti-sense | 5'-UUCUUGGCUAAAUCGCUCCTT-3' | |
| MT1F | F | 5'-GAATGTAGCAAATGGGTCAAGGTG-3′ | qRT-PCR (up) |
| | R | 5'-TCTCCTGCACCTGCGCTGGT-3′ | |
| CYP1A1 | F | 5'-TCGGCCACGGAGTTTCTTC-3′ | qRT-PCR (up) |
| | R | 5'- GGTCAGCATGTGCCCAATCA-3′ | |
| HMOX1 | F | 5’-ATTTCAGAAGGGCCAGGTGA-3′ | qRT-PCR (up) |
| | R | 5’-GGAAGTAGACAGGGGCGAAGA-3′ | |
| AJUBA | F | 5’- GATGCGGGAGCCAGAGG-3′ | qRT-PCR (up) |
| | R | 5’-CACAAGAGCAGCAAACAAAGC-3′ | |
| IL6 | F | 5’-GACCGAAGGCGCTTGTGGA-3′ | qRT-PCR (up) |
| | R | 5’-CTCATTCTGCCCTCGAGCC -3′ | |
| SLC7A11 | F | 5'-TTACCAGCTTTTGTACGAGTCT-3' | qRT-PCR (up) |
| | R | 5'-GTGAGCTTGCAAAAGGTTAAGA-3' | |
| MKNK2 | F | 5'-GTTCGAAGATGTCTATCAGC-3' | qRT-PCR (up) |
| | R | 5'-TTCTAGAACATTCCTATGTCCC -3' | |
| CYP1B1 | F | 5'-CTCTCTGGAGAAATGGCCGA-3' | qRT-PCR (up) |
| | R | 5'-AAAGGAAATCAAGCGCCACC-3' | |
| FABP5 | F | 5'-GCATTGGTTCAGCATCAG-3' | qRT-PCR (up) |
| | R | 5'-ATCCGAGTACAGGTGACA-3' | |
| CD55 | F | 5'-TGACTGTGGCCTTCCCCCAGAT-3' | qRT-PCR (up) |
| | R | 5'-GTGTTACATGAGAAGGAGATGG-3' | |
| APLN | F | 5'-AAGGCACCATCCGATACCTG-3' | qRT-PCR (down) |
| | R | 5'-ATGGGACCCTTGTGGGAGA-3' | |
| FADS2 | F | 5'-TGGAGCAGTCCTTCTTCAACG-3' | qRT-PCR (down) |
| | R | 5'-AGCCACAGCTTCCCAGACTTC-3' | |
| ACSS2 | F | 5'-GGATTCCAGCTGCAGTCTTC-3' | qRT-PCR (down) |
| | R | 5'-CAGCCAGCTCCTTCAGGTT-3' | |

| | | | |
|---|---|---|---|
| CCL2 | F | 5'-CCGAGAGGCTGAGACTAACC-3' | qRT-PCR (down) |
| | R | 5'-CTTTCATGCTGGAGGCGAGA-3' | |
| STC1 | F | 5'-TTCTGGTGCTGGTGATCAGTG-3' | qRT-PCR (down) |
| | R | 5'-TTTGGGCACAGTGGTCTGTCT-3' | |
| NDRG1 | F | 5'-CTGCACCTGTTCATCAATGC-3' | qRT-PCR (down) |
| | R | 5'-AGAGAAGTGACGCTGGAACC-3' | |
| SCD | F | 5'-TCTAGCTCCTATACCACCACCA-3' | qRT-PCR (down) |
| | R | 5'-TCGTCTCCAACTTATCTCCTCC-3' | |
| LRP5 | F | 5'-AGGCCCTACATCATTCGAGG-3' | qRT-PCR (down) |
| | R | 5'-GGGGTCTGAGTCCGAATTCA-3' | |
| EGR1 | F | 5'-CTGCGACATCTGTGGAAGAAA-3' | qRT-PCR (down) |
| | R | 5'-TGTCTGCTTTCTTGTCCTTCTG-3' | |
| CD24 | F | 5'-CCAACTAATGCCACCACCA-3' | qRT-PCR (down) |
| | R | 5'-GACGTTTCTTGGCCTGAGTC-3' | |

Abbreviations: F: Forward; R: Reverse; qRT-PCR: quantitative real-time PCR; si-RNA: Small interfering-RNA;
